# Supplementary material for: What to Do When Accumulated Exposure Affects Health but Only Its Duration Was Measured? A Case of Linear Regression
Source: Int J Environ Res Public Health. 2019 May 29;16(11):1896. doi: 10.3390/ijerph16111896 (PMC6603749; doi:10.3390/ijerph16111896)

**Supplemental Materials 3: Analysis of synthetic data with value of k inverted compared to that presented in main text.**

**Figure S1.** Adjusted estimates of β_1_ with different degrees of knowledge about joint distribution of duration and intensity of exposure when ρ = -0.5 and k=1/2.6 in four simulations of synthetic example; naïve estimate (NV) is contrasted with adjusted estimates obtained under “well-calibrated” priors on (ρ,k) that are “wide” (PR1), “narrow” (PR2) and estimates obtained with ρ and k known (KNW; the best one can do without complete data), and complete data on intensity and duration (CMP); true value is denoted by dotted line, solid lines represent 95% credible intervals; of see text for details.

**
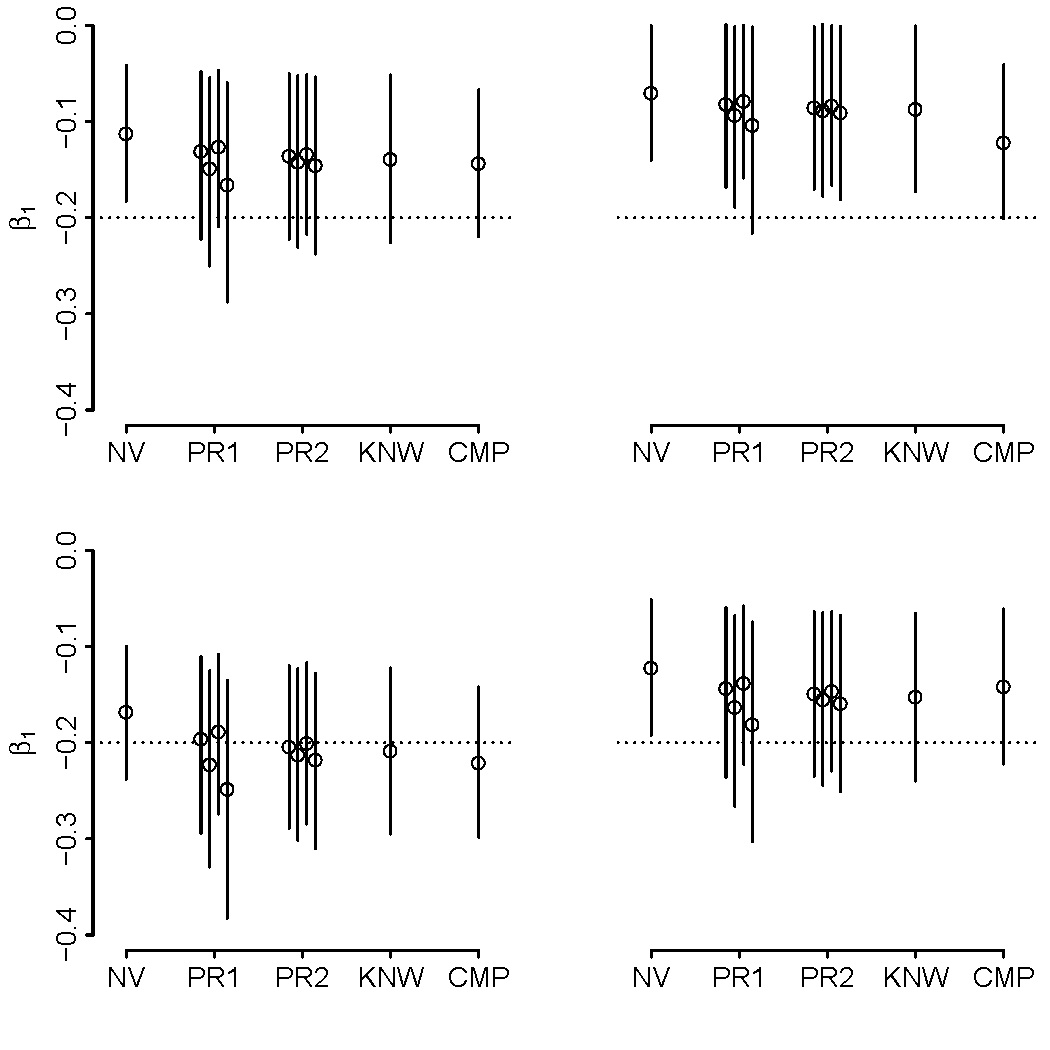
**

**Figure S2.** Adjusted estimates of β_1_ with different degrees of knowledge about joint distribution of duration and intensity of exposure when ρ = +0.5 k=1/2.6 in four simulations of synthetic example; naïve estimate (NV) is contrasted with adjusted estimates obtained under “well-calibrated” priors on (ρ,k) that are “wide” (PR1), “narrow” (PR2) and estimates obtained with ρ and k known (KNW; the best one can do without complete data), and complete data on intensity and duration (CMP); true value is denoted by dotted line, solid lines represent 95% credible intervals; of see text for details.


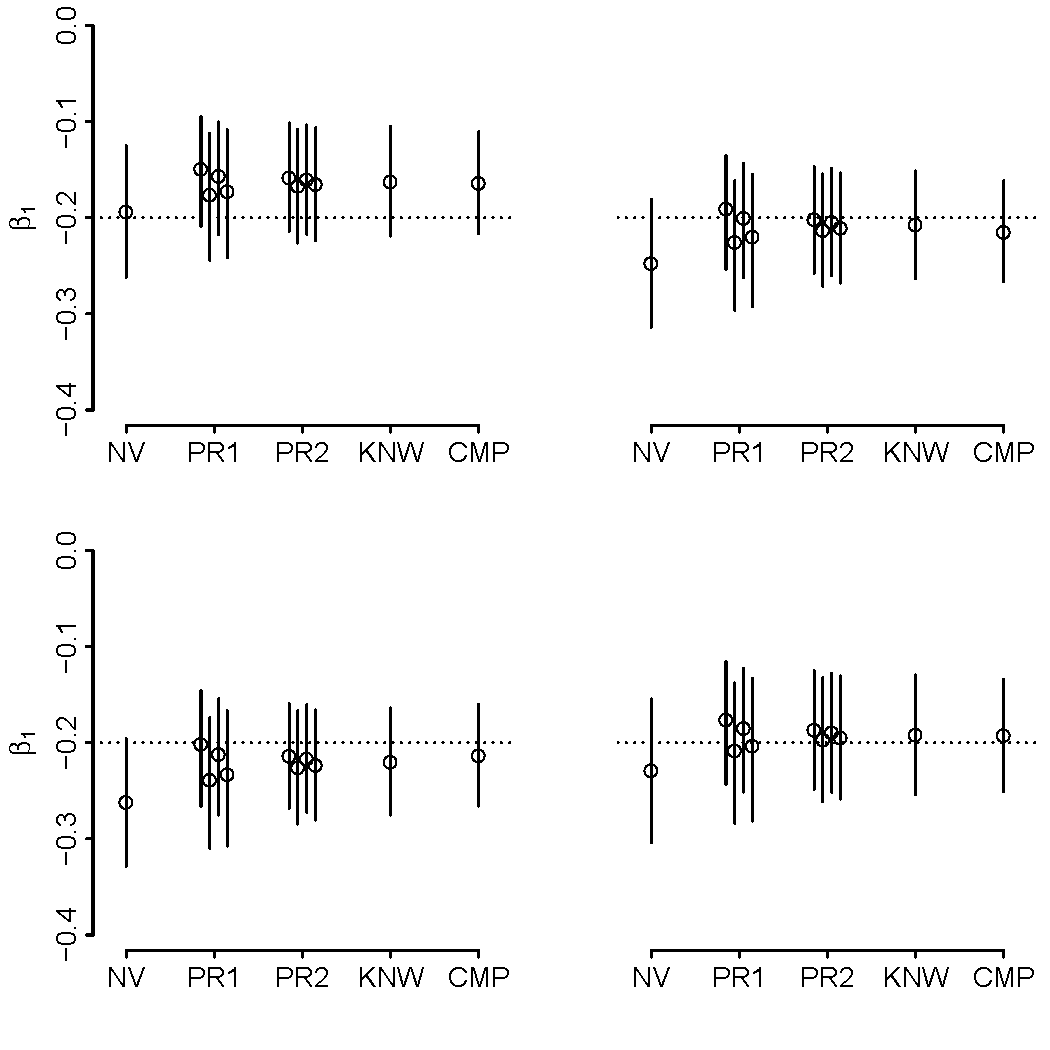

Supplement: Supplementary file 1 [file ijerph-16-01896-s001.zip › SM3.docx]
