# Supplementary material for: What to Do When Accumulated Exposure Affects Health but Only Its Duration Was Measured? A Case of Linear Regression
Source: Int J Environ Res Public Health. 2019 May 29;16(11):1896. doi: 10.3390/ijerph16111896 (PMC6603749; doi:10.3390/ijerph16111896)
Supplement: Supplementary file 1 [file ijerph-16-01896-s001.zip › SM4.docx]

# Supplemental Materials 4: Real-world Application

**Figure S3.** US NHANES 2011-2012 data on the effect of exposure to cigarette smoke on forced vital capacity (FVC) of the lungs among male current smokers (570 persons); lines in the bottom panel represent fitted linear regression models; sampling weights not applied.


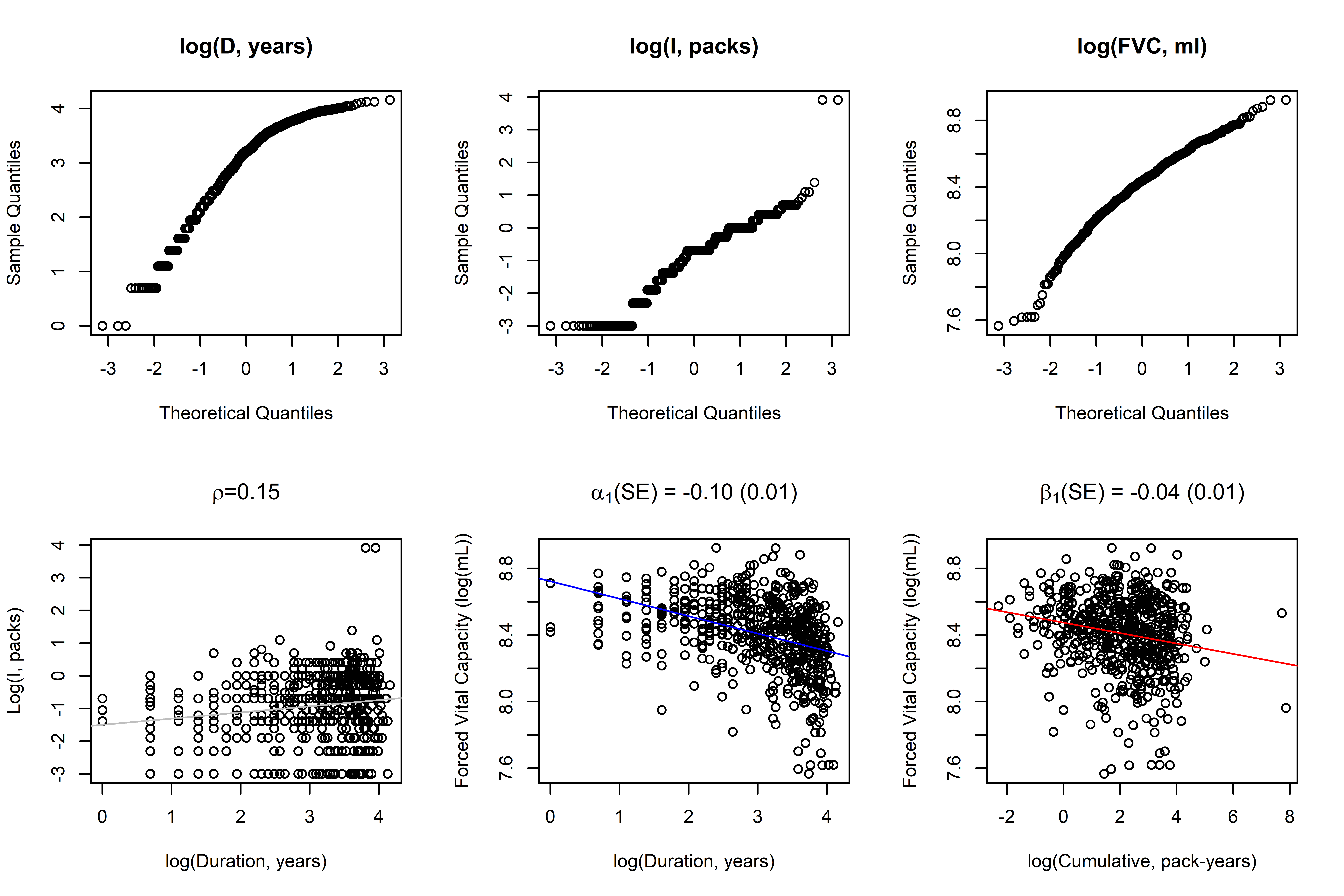


## R-code used to download, select, and analyze NHANES data and to create Figures 6 and S3.

library(RNHANES)

#############################################################################################

#PRIOR elucidation START

#2009-2010 wave of NHANES to elicit priors on rho and k and text code

#exposures

#https://wwwn.cdc.gov/Nchs/Nhanes/2009-2010/SMQ_F.htm

#SMD030 "Age started smoking cigarettes regularly"

#SMD650 "Avg # cigarettes/day during past 30 days"

#SMQ040 "Do you now smoke cigarettes?"

#SMQ020 "Smoked at least 100 cigarettes in life == 1=YES"

#RIDAGEYR age in years

#RIAGENDR 1==Male

d2010x<- nhanes_load_data("SMQ", "2009-2010", demographics=TRUE)

#select only variables of interest

d2010x<-subset(d2010x, select=c("SMD030", "SMD650", "SMQ040", "SMQ020", "RIAGENDR", "RIDAGEYR", "SEQN"))

#select male current smokers <80 years of age (spiromentry ages 6-79) & duration > 1 year

d2010x<-subset(d2010x, SMQ020==1 & RIAGENDR==1 & SMD030<80 & SMQ040<3 & RIDAGEYR<80 & RIDAGEYR>SMD030)

#outcome

#https://wwwn.cdc.gov/Nchs/Nhanes/2009-2010/SPX_F.htm

#SPXNFVC: Baseline 1st Test Spirometry, Forced Vital Capacity, in mL.

#Spirometry - Pre and Post-Bronchodilator (SPX_F)

d2010y<- nhanes_load_data("SPX_F", "2009-2010", demographics=TRUE)

d2010y<-subset(d2010y, select=c("SPXNFVC", "SEQN"))

#combine exposure and outcome

d2010xy<-merge(d2010y,d2010x, by="SEQN", all=FALSE)

d2010xy<-na.omit(d2010xy) #removes any case/row from the data frame with missing data, NA

#calculate D and C

#duration (yrs)

dur=(d2010xy$RIDAGEYR-d2010xy$SMD030)

#intenstity

int=d2010xy$SMD650/20

#priors

rho<-cor(log(dur), log(int)) #0.12

sd(log(int)) #0.971

sd(log(dur)) #0.807

k=sd(log(int))/sd(log(dur)) #1.20

rock= k*rho #0.1448542

#######end prior elucidation##############

########################################################################################

########################################################################################

#data analysis of which will be infused with prior information

#2011-2012

#EXPOSURE

d2012<- nhanes_load_data("SMQ", "2011-2012", demographics=TRUE)

d2012<-subset(d2012, select=c("SMD030", "SMD650", "SMQ040", "SMQ020", "RIAGENDR", "RIDAGEYR", "SEQN"))

#select male current smokers <80 years of age (spiromentry ages 6-79) & duration > 1 year

d2012x<-subset(d2012, SMQ020==1 & RIAGENDR==1 & SMD030<80 & SMQ040<3 & RIDAGEYR<80 & RIDAGEYR>SMD030)

#OUTCOME source data

#SPXNFVC: Baseline 1st Test Spirometry, Forced Vital Capacity, in mL.

#Spirometry - Pre and Post-Bronchodilator (SPX_F)

d2012y<- nhanes_load_data("SPX_G", "2011-2012", demographics=TRUE)

d2012y<-subset(d2012y, select=c("SPXNFVC", "SEQN"))

#combine exposure and outcome

d2012xy<-merge(d2012y,d2012x, by="SEQN", all=FALSE)

d2012xy<-na.omit(d2012xy) #removes any case/row from the data frame with missing data, NA

#calculate outcome

y<-log(d2012xy$SPXNFVC)

#calculate exposure variables

DD=(d2012xy$RIDAGEYR-d2012xy$SMD030)

d=log(DD)

II=d2012xy$SMD650/20 #packs

i=log(II)

CC=DD*II #pack-years

c=log(CC)

######################end of preparation of 2011-2012 data

########################################################################################

########################################################################################

#presentation

setwd("C:/ ")

png("FigureS3.png", units="px", width=9600, height=6400, res=1200)

par(mfrow=c(2,3))

qqnorm(d, main = "log(D, years)",

xlab = "Theoretical Quantiles", ylab = "Sample Quantiles")

qqnorm(i, main = "log(I, packs)",

xlab = "Theoretical Quantiles", ylab = "Sample Quantiles")

qqnorm(y, main = "log(FVC, ml)",

xlab = "Theoretical Quantiles", ylab = "Sample Quantiles")

plot(d, i, xlab="log(Duration, years)", ylab="Log(I, packs)", main=expression(paste(rho, "=0.15")))

abline(a= -1.49462, b=0.18885, col="grey")

plot(d, y, xlab="log(Duration, years)", ylab="Forced Vital Capacity (log(mL))", main=expression(paste(alpha[1],"(SE) = -0.10 (0.01)")))

abline(a= 8.72268, b=-0.10457, col="blue")

plot(c, y, xlab="log(Cumulative, pack-years)", ylab="Forced Vital Capacity (log(mL))", main=expression(paste(beta[1],"(SE) = -0.04 (0.01)")))

abline(a=8.474768, b=-0.031328, col="red")

dev.off()

########################################################################################

########################################################################################

##############Bayesian analysis

##############Elements of Appendix 3 are repeated for convenience

########################################################################################

########################################################################################

############################start definition of functions#####

##############################################################

#Function to set scaled beta distribution

#for prior on correlation of log(D) and log(I)

#mn=mean

#sd-standard deviation

rscbeta <- function(n, mn, sd) {

if (sd==0) {

opt <- rep(mn,n)

} else {

sd.b <- sd/2

mn.b <- (1+mn)/2

tmp <- mn.b*(1-mn.b)/sd.b^2 - 1

opt <- -1 + 2*rbeta(n, tmp*mn.b, tmp*(1-mn.b))

}

opt

}

##############################################################

##############################################################

### simple Bayesian linear regression, adapated from Hoff's book

Hoff <- function(dsm,y, n.mc=25000) {

n <- length(y)

### hyperparameters

g <- n; nu0 <- 1; s20 <- 1

Hg <- (g/(g+1)) * dsm %*% solve(t(dsm)%*%dsm)%*%t(dsm)

SSRg <- t(y)%*%(diag(1, nrow=n) - Hg) %*% y

Vb <- g * solve(t(dsm)%*%dsm)/(g+1)

Eb <- Vb%*%t(dsm)%*%y

s2 <- 1/rgamma(n.mc, (nu0+n)/2, (nu0*s20+SSRg)/2)

E <- matrix(rnorm(n.mc*2, 0, sqrt(s2)), n.mc,2)

bt.smp <- t( t(E%*%chol(Vb)) +c(Eb))

list(s2=s2, bt=bt.smp)

}

fitmod <- function(d, y, lgk.mn, lgk.sd, rho.mn, rho.sd, n.mc=25000) {

### fit y|d regression

ftA <- Hoff(cbind(1,d), y, n.mc=n.mc)

### fit d (just variance)

var.d.smp <- 0.5*sum(y^2)/(n+1)/rgamma(n.mc, 0.5*(n+1))

### sample from rho,k prior

rho.smp <- rscbeta(n.mc, rho.mn, rho.sd)

k.smp <- exp(rnorm(n.mc, lgk.mn, lgk.sd))

### extract the posterior samples we need

b1.smp <- ftA$bt[,2]/(1+rho.smp*k.smp)

var.y.d.smp <- ftA$s2

### which Monte Carlo draws satisfy constaint?

ind <- b1.smp^2 < ( var.y.d.smp / ( (1-rho.smp)^2 * k.smp^2 * var.d.smp ) )

### output

list(b1=b1.smp[ind], b1.excld=b1.smp[!ind], prop.out=mean(!ind))

}

############################end definition of functions###############

#######################################################################

#priors on correlation (rho) and k

#######################################################################

### wide priors (Priors 1)

rho.sd.wide <- .2

lgk.sd.wide <- .125

## narrow priors (Prior 2)

rho.sd.nrw <- rho.sd.wide/3

lgk.sd.nrw <- lgk.sd.wide/3

#######################################################################

#Analysis

#values of rho and k are drawn from analysis of 2009-2010 NHANES data

#######################################################################

n <- length(y)

### complete data answer when both log(D) and log(I) are observed

ans.cmplt <- Hoff(cbind(1,d+i), y)$bt[,2]

### naive answer when only log(D) is known

ans.naive <- Hoff(cbind(1,d), y)$bt[,2]

### FIXED rho,k answer #change label from Appendix 3 as these are just point guess, we pretend not to know 2011-2012 intensity

tmp <- fitmod(d, y,

lgk.mn=log(k), lgk.sd=0,

rho.mn=rho, rho.sd=0)

ans.known <- tmp$b1; pro.known <- tmp$prop.out

### first set of priors (#1, wider)

rho.sd <- rho.sd.wide; lgk.sd <- lgk.sd.wide

ans.1 <- list(); pro.1 <- rep(NA,4)

tmp <- fitmod(d,y,

rho.mn=rho+rho.sd, rho.sd=rho.sd,

lgk.mn=log(k)+lgk.sd, lgk.sd=lgk.sd)

ans.1[[1]] <- tmp$b1; pro.1[1] <- tmp$prop.out

tmp <- fitmod(d,y,

rho.mn=rho-rho.sd, rho.sd=rho.sd,

lgk.mn=log(k)-lgk.sd, lgk.sd=lgk.sd)

ans.1[[2]] <- tmp$b1; pro.1[2] <- tmp$prop.out

tmp <- fitmod(d,y,

rho.mn=rho+rho.sd, rho.sd=rho.sd,

lgk.mn=log(k)-lgk.sd, lgk.sd=lgk.sd)

ans.1[[3]] <- tmp$b1; pro.1[3] <- tmp$prop.out

tmp <- fitmod(d,y,

rho.mn=rho-rho.sd, rho.sd=rho.sd,

lgk.mn=log(k)+lgk.sd, lgk.sd=lgk.sd)

ans.1[[4]] <- tmp$b1; pro.1[4] <- tmp$prop.out

### second set of priors (#2, narrower)

rho.sd <- rho.sd.nrw; lgk.sd <- lgk.sd.nrw

ans.2 <- list(); pro.2 <- rep(NA,4)

tmp <- fitmod(d,y,

rho.mn=rho+rho.sd, rho.sd=rho.sd,

lgk.mn=log(k)+lgk.sd, lgk.sd=lgk.sd)

ans.2[[1]] <- tmp$b1; pro.2[1] <- tmp$prop.out

tmp <- fitmod(d,y,

rho.mn=rho-rho.sd, rho.sd=rho.sd,

lgk.mn=log(k)-lgk.sd, lgk.sd=lgk.sd)

ans.2[[2]] <- tmp$b1; pro.2[2] <- tmp$prop.out

tmp <- fitmod(d,y,

rho.mn=rho+rho.sd, rho.sd=rho.sd,

lgk.mn=log(k)-lgk.sd, lgk.sd=lgk.sd)

ans.2[[3]] <- tmp$b1; pro.2[3] <- tmp$prop.out

tmp <- fitmod(d,y,

rho.mn=rho-rho.sd, rho.sd=rho.sd,

lgk.mn=log(k)+lgk.sd, lgk.sd=lgk.sd)

ans.2[[4]] <- tmp$b1; pro.2[4] <- tmp$prop.out

########################################################################

### what proportion of sampled points discarded due to constraint?

print(pro.1)

print(pro.2)

print(pro.known)

##########################################################################

### Examine distribution of samples from posterior distribution of beta 1

###estimates interms of 95% Credible Interval and Median

#Only log(D), naive

quantile(ans.naive, c(.025,0.5, .975))

#Priors 1

quantile(ans.1[[1]], c(.025,0.5, .975))

quantile(ans.1[[2]], c(.025,0.5, .975))

quantile(ans.1[[3]], c(.025,0.5, .975))

quantile(ans.1[[4]], c(.025,0.5, .975))

#Priors 2

quantile(ans.2[[1]], c(.025,0.5, .975))

quantile(ans.2[[2]], c(.025,0.5, .975))

quantile(ans.2[[3]], c(.025,0.5, .975))

quantile(ans.2[[4]], c(.025,0.5, .975))

#FIXED (rho, k)

quantile(ans.known, c(.025, 0.5, .975))

#complete data on D and I

quantile(ans.cmplt, c(.025, 0.5, .975))

#################PLOT RESULTS start

lbls <- c("Naive", "Prior 1", "Prior 2", "Fixed","Complete")

yl <- c(min(quantile(ans.naive,.025),

quantile(ans.known,.025),

quantile(ans.cmplt,.025),

unlist(lapply(ans.1, quantile, .025)),

unlist(lapply(ans.2, quantile, .025))),

max(quantile(ans.naive,.975),

quantile(ans.known,.975),

quantile(ans.cmplt,.975),

unlist(lapply(ans.1, quantile, .975)),

unlist(lapply(ans.2, quantile, .975))))

yl[1] <- floor(10*yl[1]-1)/10

yl[2] <- ceiling(10*yl[2])/10

setwd("C:/ ")

png("Figure6.png", units="px", width=9600, height=6400, res=1200)

plot(-1,-2, xlim=c(0.8,5.2),ylim=yl, xlab="",ylab=expression(beta[1]), xaxt="n")

abline(h=quantile(ans.cmplt,.025), lty=3, lwd=3) ### indicates 2.5 pctl with complete data

text(1:5, yl[1]+.05, lbls)

points(1, mean(ans.naive))

points(rep(1,2), quantile(ans.naive, c(.025,.975)), type="l")

eps <- .1

for (i in 1:4) {

points(2+eps*(i-2.5), mean(ans.1[[i]]))

points(rep(2+eps*(i-2.5),2), quantile(ans.1[[i]], c(.025,.975)), type="l")

}

for (i in 1:4) {

points(3+eps*(i-2.5), mean(ans.2[[i]]))

points(rep(3+eps*(i-2.5),2), quantile(ans.2[[i]], c(.025,.975)), type="l")

}

points(4, mean(ans.known))

points(rep(4,2), quantile(ans.known, c(.025,.975)), type="l")

points(5, mean(ans.cmplt))

points(rep(5,2), quantile(ans.cmplt, c(.025,.975)), type="l")

dev.off()

#################PLOT RESULTS END
